# Zusammensetzung von Schockraumteams: Gelebte Realität in 12 überregionalen Traumazentren

**DOI:** 10.1007/s00113-024-01532-z

**Published:** 2025-02-13

**Authors:** Vera Pedersen, Christian Waydhas, Valentin Clemens, Orkun Özkurtul, Lisa Hackenberg, Tristan Pfläging, Rolf Lefering, André Nohl, Uwe Schweigkofler, Matthias Fröhlich, Fabian Laue, Markus Baacke, Philipp Störmann, Helena Düsing, Kai Sprengel, Thomas Paffrath, Kai Oliver Jensen, Philipp Faul, Tobias Ahnert, Sebastian Imach, Christian Kleber, Anette Keß, Dan Bieler, Heiko Trentzsch, Studiengruppe NIS Trauma-Team Activation and Trauma/Injury Care (NIS-TAcTIC), Lars Becker, Sascha Flohe, Stefan Huber-Wagner, Oliver Kamp, Marion Laumann, Carsten Mand, Gerrit Matthes, Frithjof Wagner, Bernd Wohlrath

**Affiliations:** 1 Klinik und Poliklinik für Orthopädie und Unfallchirurgie, Muskuloskelettales Universitätszentrum München (MUM), LMU Klinikum, Marchioninistr. 15, 81377 München, Deutschland; 2 Integriertes Notfallzentrum (INZ), Universitätsmedizin Mannheim UMM, Universitätsklinikum Mannheim GmbH, Theodor-Kutzer-Ufer 1–3, 68167 Mannheim, Deutschland; 3 Klinik und Poliklinik für Orthopädie, Unfallchirurgie und Plastische Chirurgie, Universitätsklinikum Leipzig, Leipzig, Deutschland; 4 Klinik für Orthopädie und Unfallchirurgie, Klinikum Dritter Orden, München, Deutschland; 5 Klinik für Unfallchirurgie und Orthopädie, Wiederherstellungs- und Handchirurgie, Verbrennungsmedizin, Bundeswehrzentralkrankenhaus Koblenz, Koblenz, Deutschland; 6 Klinik für Orthopädie und Unfallchirurgie, Universitätsklinikum Düsseldorf, Medizinische Fakultät, Heinrich-Heine-Universität Düsseldorf, Düsseldorf, Deutschland; 7 Institut für Forschung in der Operativen Medizin (IFOM), Universität Witten/Herdecke, Köln, Deutschland; 8 Zentrum für Notfallmedizin, BG Klinikum Duisburg, Duisburg, Deutschland; 9 Abteilung für Orthopädie und Unfallchirurgie, BG Unfallklinik Frankfurt, Frankfurt am Main, Deutschland; 10 Klinik für Orthopädie, Unfallchirurgie und Sporttraumatologie, Universität Witten/Herdecke, Köln-Merheim Medical Center (CMMC), Köln, Deutschland; 11 Klinik für Unfall- und Wiederherstellungschirurgie, Klinikum Ernst von Bergmann, Potsdam, Deutschland; 12 Abteilung für Unfall- und Wiederherstellungschirurgie, Krankenhaus der Barmherzigen Brüder Trier, Trier, Deutschland; 13 Klinik für Unfallchirurgie, Wiederherstellungschirurgie, Handchirurgie, Universitätsklinikum Frankfurt, Frankfurt, Deutschland; 14 Klinik für Unfall‑, Hand und Wiederherstellungschirurgie, Universitätsklinikum Münster, Münster, Deutschland; 15 Hirslanden Klinik St. Anna, Praxis medOT, Luzern, Schweiz; 16 Klinik für Traumatologie, Universitätsspital Zürich, Zürich, Schweiz; 17 Klinik für Unfall‑, Hand- und Wiederherstellungschirurgie, Universitätsklinikum Essen, Universität Duisburg-Essen, Essen, Deutschland; 18 Abteilung Unfall- und Handchirurgie, Cellitinnen-Severinsklösterchen, Krankenhaus der Augustinerinnen, Köln, Deutschland; 19 Institut für Notfallmedizin und Medizinmanagement (INM), Klinikum der Universität München, München, Deutschland

**Keywords:** Schockraumteam, Schockraumalarmierungskriterien, Polytrauma, Mortalität, Fachdisziplinen, Trauma team, Trauma team activation criteria, Severely injured, Mortality, Specialty

## Abstract

**Hintergrund:**

Die Bereitstellung spezialisierter Schockraumteams zur Schwerverletztenversorgung ist nach den Vorgaben der S3-Leitlinie Polytrauma/Schwerverletztenversorgung der AMWF obligat und die Zusammensetzung durch das *Weißbuch Schwerverletztenversorgung* festgelegt. In jeder Versorgungsstufe wird das Basisteam aus den 4 Disziplinen Orthopädie und Unfallchirurgie, Anästhesie, Radiologie und der Notfallpflege der Notaufnahme zusammengesetzt, mit weiteren Anpassungen je nach Versorgungsstufe des Krankenhauses. Ziel der vorliegenden Studie ist die Untersuchung der gelebten Realität bei der Zusammensetzung der Schockraumteams.

**Methodik:**

Bei der prospektiven, multizentrischen Beobachtungsstudie wurden in 12 überregionalen Traumazentren in Deutschland und der Schweiz insgesamt 3753 Patienten nach Unfällen in der Notaufnahme behandelt, darunter 964 Patienten (26 %) nach vorangegangener Schockraumalarmierung.

**Ergebnisse:**

In 94,7 % der Schockraumversorgungen waren alle 4 der geforderten Disziplinen anwesend; im Durchschnitt waren 6 Personen an der Schockraumversorgung beteiligt. Die 48-h-Mortalität betrug 3 % der über den Schockraum versorgten Patienten; bei allen verstorbenen Patienten waren *während* der Schockraumversorgung alle 4 Disziplinen anwesend. Bei Patienten mit mindestens einem Alarmierungskriterium der Kategorie A waren bei 97,7 % der Versorgung ein vollständiges Team aus 4 Disziplinen an der Versorgung beteiligt.

**Diskussion:**

In fast 98 % der Fälle, in denen Alarmierungskriterien der Kategorie A vorliegen, sind alle 4 der im Weißbuch geforderten Disziplinen zur Patientenversorgung im Schockraum anwesend. Dies geht mit einer mittleren Ressourcenbindung von 6,6 Personen einher. Das Fehlen einer oder mehrerer Disziplinen bei der Schockraumversorgung scheint die frühe Mortalität der Schwerverletzten nicht signifikant zu beeinflussen.

**Graphic abstract:**

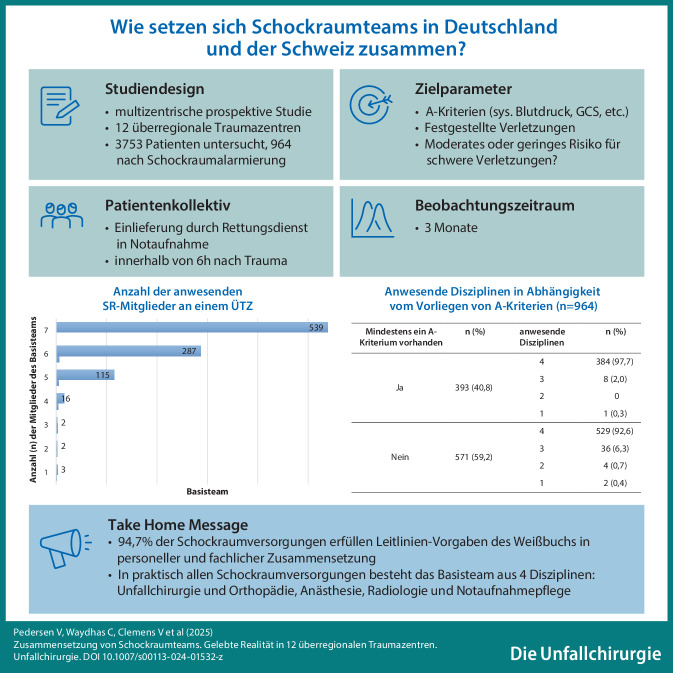

## Abstract (en)

**Background:**

The provision of specialized trauma teams for the care of severely injured patients is mandatory according to the requirements of the S3 guidelines polytrauma and the composition is determined by the White Book Medical Care of the Severely Injured (*Weißbuch Schwerverletztenversorgung*). In each level of care the basic resuscitation room team is composed of four disciplines: orthopedics and trauma surgery, anesthesia, radiology and emergency medicine in the emergency department.

**Material and methods:**

A prospective, multicenter observational study was conducted in 12 supraregional trauma centers in Germany and Switzerland, where a total of 3753 patients were treated in the emergency department following accidents. Amongst them 964 patients (26%) were treated after prior trauma team activation.

**Results:**

In 94.7% of the trauma room care instances all 4 required disciplines were present, with an average of 6.6 individuals involved in the trauma room care. The 48‑h mortality rate was 3% among patients receiving trauma room care. In all deceased patients, all four disciplines were present during the trauma room care. At least one or more high-risk criteria for serious injuries were present in 40.8% of the patients. In these cases, a complete team consisting of all 4 disciplines was involved in 97.7% of the care instances.

**Conclusion:**

In nearly 98% of cases where high-risk criteria for serious injuries (category A activation criteria) all 4 required disciplines were present in the trauma room for patient care. This was associated with an average resource commitment of 6.6 individuals. The absence of one or more disciplines in trauma room care does not appear to significantly affect early mortality in the severely injured.

Die fachliche und personelle Zusammensetzung der bei der Schwerverletztenversorgung eingesetzten Teams ist, in Abhängigkeit von der Versorgungsstufe, durch das *Weißbuch Schwerverletztenversorgung* festgelegt und muss in Verfahrensanweisungen standortspezifisch definiert werden. Systematische Untersuchungen über die gelebte Realität der Vorgaben im TraumaNetzwerk DGU® (Deutsche Gesellschaft für Unfallchirurgie) und deren Auswirkung auf das Überleben und das funktionelle Outcome der Schwerverletzten liegen bislang nicht vor.

In der aktuellen Version des *Weißbuch Schwerverletztenversorgung* der Deutschen Gesellschaft für Unfallchirurgie (DGU®) [9] wird u. a. die Ausstattung von Traumazentren definiert. Die personelle und fachliche Zusammensetzung der Basisteams für die Schockraumversorgung wird in Abhängigkeit von der Versorgungsstufe, d. h. lokales (LTZ), regionales (RTZ) oder überregionales Traumazentrum (ÜTZ), festgelegt. In der aktuellen Version (Update 2022) der „S3-Leitlinie Polytrauma/Schwerverletzten-Behandlung“ [7] werden mit einem hohen Empfehlungsgrad feste Teams (sog. Schockraumteams), die nach vorstrukturierten Plänen arbeiten und/oder ein spezielles Training absolviert haben [13], empfohlen. Die personelle Zusammensetzung der Teams wird interdisziplinär und multiprofessionell gefordert [9].

Das Schockraumbasisteam in einem ÜTZ wird in den verschiedenen Auflagen des *Weißbuch Schwerverletztenversorgung* [9, 10] sowie den S3-Polytraumaleitlinien 2016 [4, 8] unterschiedlich definiert. Zum Zeitpunkt der Datenerhebung war die neue S3-Leitlinie 2022 in Überarbeitung.

Während im Weißbuch des Jahres 2012 (2. Auflage) [10] für das „Basisteam“ eines ÜTZ noch 8 Mitglieder gefordert wurden, sind dies in der 3. Auflage des Jahres 2019 [9] nur noch 7 Mitglieder, da die Präsenz des Radiologen im Schockraum nicht mehr als erforderlich angesehen wurde. So wurden für das Basisteam in einem ÜTZ ein Facharzt (bzw. Facharztstandard) für Orthopädie und Unfallchirurgie, ein Weiterbildungsassistent in Orthopädie und Unfallchirurgie (oder Weiterbildungsassistent in Viszeralchirurgie oder Allgemeinchirurgie), ein Facharzt für Anästhesiologie (bzw. Facharztstandard), 2 Pflegekräfte Notaufnahme, eine Pflegekraft Anästhesiologie und eine medizinisch-technische Radiologiefachkraft (MTRA) vorgeschrieben, in der Summe also 7 Mitglieder [9]. Dies entspricht auch den Ausführungen in der S3-Polytrauma-Leitline des Jahres 2016 [4, 8]. Wegen der steigenden Inanspruchnahme der Ressource „Schockraum“ mit entsprechendem personellen und logistischen Aufwand auch bei Patienten, die diese Ressource in der rückblickenden Betrachtung nicht benötigt hätten, ist die Reevaluation der Schockraumkriterien erforderlich [3, 13].

Im *Weißbuch Schwerverletztenversorgung* des Jahres 2019 wurde außerdem, im Sinne einer bedarfsgerechten Schockraumaktivierung mit einer effektiven Ressourcenmobilisation in ÜTZ, die Möglichkeit eines „angepassten Basisteams“ eingeführt: Dieses soll initial im Schockraum präsent sein und ggf. die weiteren Eskalationsstufen aktivieren. Das angepasste Schockraumbasisteam soll mindestens einen Facharzt für Orthopädie und Unfallchirurgie bzw. Facharzt für Chirurgie (Facharztstandard) (ATLS® oder ETC® geschult), einen Facharzt für Anästhesie (Facharztstandard), eine Pflegefachkraft Notaufnahme, eine Pflegefachkraft Anästhesie und eine MTRA [9], also 5 Teammitglieder, umfassen. Die bedarfsangepasste Alarmierung des Basisteams setzt jedoch voraus, dass eine zuverlässige und strukturierte Übermittlung des Zustands des Patienten vom Unfallort erfolgt (Arzt-Arzt-Gespräch) und eine jederzeit erweiterbare, d. h. eskalierbare Alarmierung des gesamten Schockraumteams innerhalb kürzester Zeit sichergestellt ist.

Validierte Untersuchungen oder prospektive vergleichende Studien über die genaue personelle Zusammensetzung der Schockraumteams liegen derzeit nicht vor [13]. Die Teamzusammensetzung soll sich v. a. an den sich durch das Verletzungsmuster zu leistenden Aufgaben orientieren und die notfallchirurgische und notfallmedizinische Kompetenz abdecken [13], so die aktuelle Empfehlung der S3-Leitlinie.

Das Basisteam muss nach Weißbuch, unabhängig von der Versorgungsstufe, rund um die Uhr vorgehalten werden [9]. Dieses stellt insbesondere für RTZ und ÜTZ eine relevante Herausforderung an die Stellen- und Dienstplanungen dar und wird durch die Vorgaben der aktuellen Tarifpolitik im öffentlichen Dienst zur Arbeitszeitregelung erschwert. Nur wenige ältere Studien haben die Zusammensetzung interdisziplinärer Schockraumteams untersucht und diese beschrieben [12, 22], eine Outcome-orientierte Untersuchung hinsichtlich der Versorgungsqualität der schwer verletzten Patienten liegt derzeit nicht vor.

Ziel der vorliegenden prospektiven Studie war eine systematische Untersuchung über die gelebte Realität der Vorgaben zu fachlicher und personeller Zusammensetzung im TraumaNetzwerk DGU® und deren Auswirkung auf die Mortalität der Schwerverletzten.

## Methodik

### Studiendesign

Bei der vorliegenden Studie handelt sich um eine prospektive, multizentrische, nichtinterventionelle Querschnittkohortenstudie in 12 Traumazentren (TZ) in Deutschland und der Schweiz. Alle 12 teilnehmenden Krankenhäuser sind als überregionale Level-1-Traumazentren zertifiziert. Level-1-Traumazentren sind die TZ mit der höchsten Versorgungsstufe. Die Teilnahme war freiwillig, und die 12 Kliniken wurden im Rahmen einer Umfrage unter den Mitgliedern der Sektion Notfall‑, Intensiv- und Schwerverletztenversorgung der Deutschen Gesellschaft für Unfallchirurgie sowie teilnehmenden ÜTZ akquiriert. In jedem TZ wurden die Daten jeweils während eines 3‑monatigen Zeitraums in einem Zeitfenster zwischen Dezember 2019 und Februar 2021 erhoben. Die Studie ist im Einklang mit der Deklaration von Helsinki und ihren Änderungen und wurde gemäß den Leitlinien Strengthening the Reporting of Observational Studies in Epidemiology Statement (STROBE) durchgeführt [24]. Die Studie wurde von der federführenden Ethikkommission der Universität Leipzig (Aktenzeichen 060/18-ek) und nacheinander von allen lokalen Ethikkommissionen der beteiligten Einrichtungen genehmigt. Die schriftliche Einverständniserklärung der Patienten wurde so früh wie möglich nach der Einlieferung eingeholt. Es gab keine spezifischen Studieneingriffe, und es wurden nur Routinedaten erhoben.

### Studienpopulation

Während des Studienzeitraums wurden konsekutiv alle erwachsenen Patienten, die nach einem Trauma mit dem Rettungsdienst eingeliefert wurden, nach folgenden Kriterien erfasst:

- Einlieferung in die Notaufnahme durch den Rettungsdienst,
- jeder akute Traumamechanismus als Grund für die Aktivierung des Rettungsdienstes,
- Alter ≥ 18 Jahre,
- primäre Aufnahme innerhalb von 6 h nach Trauma.

Selbsteinweisungen und Überweisungen aus anderen Einrichtungen wurden ausgeschlossen.

### Schockraumalarmierung

Die Schockraumalarmierung (Traumateamaktivierung, TTA) wurde durch den Rettungsdienst eingeleitet. Die TTA sollte der nationalen Leitlinie für schwere Traumata unter Verwendung etablierter Feldtriagekriterien folgen [9]. Die TTA kann jedoch auch nach Ermessen des Rettungsdienstes eingeleitet (oder vermieden) werden. Die Triagekriterien sind unterteilt in Kriterien mit einem hohen Risiko für schwere Verletzungen (HRSI) (Infobox 1) und mit einem moderaten Risiko für schwere Verletzungen (MRSI). Alle Patienten, auf die keines dieser Kriterien zutrifft, wurden als Patienten mit geringem Risiko für schwere Verletzungen (LRSI) eingestuft.

#### Infobox Kriterien mit hohem Risiko für eine schwere Verletzung (Empfehlungsgrad A, sog. A-Kriterien) zur Aufnahme in den Schockraum eines TraumaZentrum DGU®

Störung der Vitalparameter:

- Systolischer Blutdruck unter 90 mm Hg nach Trauma
- GCS unter 9 nach Trauma
- Atemstörungen/Intubationspflicht nach Trauma

Festgestellte Verletzungen:

- Penetrierende Verletzungen der Rumpf‑/Hals-Region
- Schussverletzungen der Rumpf‑/Hals-Region
- Frakturen von mehr als 2 proximalen Knochen
- Instabiler Thorax
- Instabile Beckenfraktur
- Amputationsverletzung proximal der Hände/Füße
- Verletzungen mit neurologischer Querschnittssymptomatik
- Offene Schädelverletzung
- Verbrennung > 20 % von Grad ≥ 2b

Gemäß den nationalen Traumaleitlinien ist für die TTA die Anwesenheit eines Basistraumateams erforderlich, das aus mindestens 3 Ärzten (2 Chirurgen und einem Anästhesisten) besteht, von denen mindestens ein Anästhesist und ein Chirurg den Status eines Facharztes haben müssen, sowie aus je einer Pflegekraft Anästhesie und Notaufnahme und einer medizinisch-technischen Radiologiefachkraft (MTRA). Erweiterte Traumateams (z. B. Viszeralchirurgen, Neurochirurgen, Gefäßchirurgen und andere Spezialisten) müssen bereitgestellt werden und innerhalb von 20–30 min nach der Alarmierung eintreffen [9].

### Datenerhebung

Alle für diese Studie verwendeten Daten wurden auf einem zuvor definierten standardisierten Protokoll erfasst. Die Daten wurden auf standardisierten Papierfallberichtsformularen erfasst oder mithilfe der Oracle-APEX-Technologie direkt in ein webbasiertes Dateneingabeformular eingegeben. Die Dateneingabe wurde pseudonymisiert. Die webbasierte Datenbank wurde am Institut für Notfallmedizin und Medizinmanagement (INM) der Ludwig-Maximilians-Universität München gehostet. Die Daten wurden auf Plausibilität und Vollständigkeit geprüft. Der Datensatz, der zur Analyse zur Verfügung gestellt wurde, war vollständig anonymisiert.

Die Daten enthielten u. a. demografische Informationen, Informationen über den Traumamechanismus, die Art des Rettungsdienstes, präklinische Vitalparameter und Parameter bei Aufnahme, die präklinische Behandlung, Triage-Entscheidung vor Ort, TTA, Verletzungsmuster und Diagnosen, Notfalleingriffe und Operationen, stationäre Aufnahme ins Krankenhaus und auf die Intensivstation sowie die Krankenhaussterblichkeit innerhalb von 48 h.

### Statistik

Die Studie sollte mindestens 3000 Traumaeinweisungen umfassen, um eine ausreichende Stichprobengröße für Subgruppenanalysen, die im Rahmen einer zuvor erfolgten Power-Analyse errechnet wurden, zu erhalten. Auf der Grundlage der erwarteten Patientenzahl pro TZ war ein 3‑monatiger Zeitraum für die Datenerfassung pro Krankenhaus vorgesehen.

### Statistische Auswertung

Die statistische Analyse wurde mit SPSS (Version 23, IBM Inc., Armonk, NY, USA) durchgeführt. Zusätzlich zur deskriptiven Statistik wurden Chi-Quadrat-Tests für Häufigkeiten und Mann-Whitney-U-Tests für metrische und ordinale Daten verwendet. Das Signifikanzniveau ist für alle Tests auf 5 % (*p* < 0,05) festgelegt.

## Ergebnisse

### Patientenkollektiv

Insgesamt wurden 3753 Patienten in 12 Studienzentren in die Studie eingeschlossen. Davon wurden 974 (26,0 %) Patienten nach einer Schockraumalarmierung im Schockraum versorgt. Bei 10 Patienten (1,0 %) fehlten die Angaben zur Zusammensetzung des Schockraumteams. Eine Übersicht über die Demografie und das Verletzungsmuster der 964 zur weiteren Auswertung eingeschlossenen Patienten gibt Tab. 1.

**Tab. 1:** Charakteristik der im Schockraum versorgten Patienten (*n* = 964). Die Daten werden als Mittelwert (± SD), Median (mit Bereich) oder in Prozent der Patienten angegeben

*Merkmal*	
Alter (Jahre)	50,5 (± 21,3)
Geschlecht männlich	66,6 % (*n* = 642)
Injury Severity Score (ISS; Punkte)	10,6 (± 9,9)
*Verletzungsmuster (AIS >* *0)*	
AIS, Kopf	55,2 % (*n* = 532)
AIS, Thorax	31,4 % (*n* = 303)
AIS, Abdomen	8,6 % (*n* = 83)
AIS, Wirbelsäule	27,8 % (*n* = 268)
AIS, obere Extremität	29,9 % (*n* = 288)
AIS, untere Extremität	24,9 % (*n* = 240)
AIS, Becken (%)	7,7 % (*n* = 74)
*Luftgebundener Transport (%)*	27,4 % (*n* = 264)
*Unfallmechanismus*	
Stumpf	95,4 % (*n* = 920)
Penetrierend	4,6 % (*n* = 44)
*Unfallart*	
Verkehr	51,8 % (*n* = 499)
Sport	2,8 % (*n* = 27)
Sturz	37,2 % (*n* = 359)
Sonstige	3,8 % (*n* = 37)
Schuss/Stich/Gewalt	4,4 % (*n* = 42)
*Disposition*	
Stationäre Aufnahme	89,7 % (*n* = 865)
Intensivbehandlung	53,6 % (*n* = 517)
*Outcome*	
Liegedauer auf der Intensivstation (Tage) (Median (Min–Max))	2 (1–57)
Verstorben innerhalb von 48 h	3,9 % (*n* = 29)

*SD* Standardabweichung

### Teammitglieder und Disziplinen im Schockraum

**Abb. 1:**
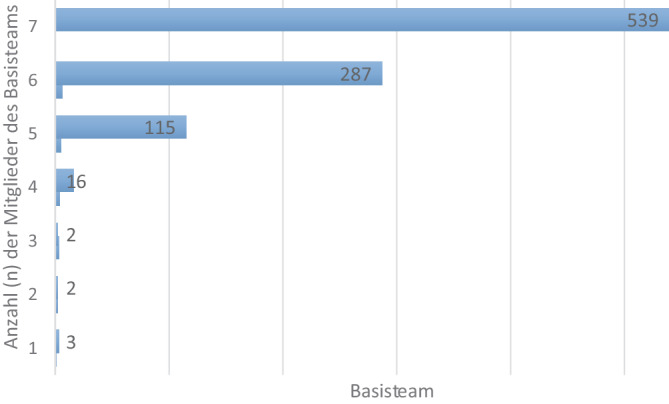
Verteilung der Anzahl der im Schockraum anwesenden Mitglieder (*n* = 964) des Basisteams eines ÜTZ. *7* bedeutet die Anwesenheit von mindestens 7 Mitgliedern des Basisteams nach Weißbuch [9]

Bei 539 von 964 (55,9 %) Schockraumversorgungen war ein vollständiges Schockraumteam von mindesten 7 Personen nach Weißbuch [9] im Schockraum anwesend (Abb. 1). Bei Anwesenheit von 7 Personen (*n* = 320) war in 238 Versorgungen (74 %) die 2. Pflegekraft der Notaufnahme nicht anwesend; in 20 Versorgungen hat die MTRA im Schockraumteam gefehlt. Im Durchschnitt waren 6,6 Personen bei der Schockraumversorgung anwesend. In 56 % der Versorgungen waren mindestens 7 der im Weißbuch geforderten Mitglieder eines Basisteams anwesend. Bezogen auf das angepasste Schockraumteam mit mindestens 5 Mitgliedern initial im Schockraum lag die Erfüllungsrate bei 97,6 % der Versorgungen.

**Abb. 2:**
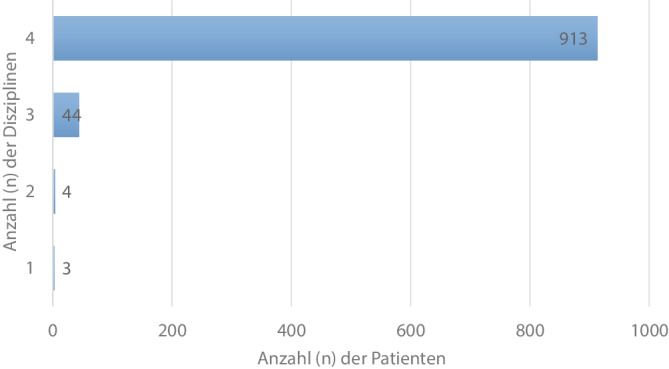
Häufigkeitsverteilung der Anzahl der im Schockraum anwesenden Disziplinen (*n* = 964). Vier Disziplinen entspricht der Anwesenheit von Orthopädie und Unfallchirurgie, Anästhesie, Radiologie und Notaufnahmepflege

Bei 94,7 % der Schockraumversorgungen waren alle 4 der geforderten Disziplinen Orthopädie und Unfallchirurgie, Anästhesie, Radiologie und Notaufnahmepflege anwesend; bei 51 (5,3 %) der über den Schockraum versorgten Patienten waren 3 oder weniger der geforderten Disziplinen anwesend (Abb. 2).

### Letalität

Insgesamt sind 29 (3 %) der über den Schockraum versorgten Patienten innerhalb von 48 h im Krankenhaus verstorben. Bei allen verstorbenen Patienten waren während der Schockraumversorgung alle 4 Disziplinen anwesend.

### Schockraumteams und Alarmierungskriterien

Bei 393 (40,8 %) Patienten lagen mindestens ein oder mehrere Alarmierungskriterien der Kategorie A (hohes Risiko für eine schwerwiegende Verletzung) [9] vor. Die Tab. 2 zeigt die Anzahl der bei diesen Patienten anwesenden Disziplinen bei der Schockraumversorgung, verglichen mit Patienten ohne A‑Kriterien (59,2 %).

**Tab. 2:** Anwesende Disziplinen in Abhängigkeit vom Vorliegen von A‑Kriterien [9] für die Schockraumalarmierung (*n* = 964)

Mindestens ein A‑Kriterium vorhanden	*n* (%)	Anwesende Disziplinen	*n* (%)
Ja	393 (40,8)	4	384 (97,7)
		3	8 (2,0)
		2	0
		1	1 (0,3)
Nein	571 (59,2)	4	529 (92,6)
		3	36 (6,3)
		2	4 (0,7)
		1	2 (0,4)

In 15 Fällen (1,6 %) war initial weder ein Facharzt noch ein Assistenzarzt der Orthopädie und Unfallchirurgie anwesend, d. h. initial nur ein Facharzt der Anästhesie, davon wurde in 6 Fällen (0,9 %) die Orthopädie und Unfallchirurgie nachalarmiert. In 18 Fällen (1,9 %) war die Anästhesie initial nicht anwesend (d. h. nur Orthopädie und Unfallchirurgie anwesend); in 3 Fällen wurde die Anästhesie nachalarmiert. In 45 Fällen (4,7 %) war die Radiologie initial nicht an der Schockraumversorgung beteiligt und wurde in 18 (1,9 %) Fällen nachalarmiert. Bei keiner Schockraumversorgung waren weder die Orthopädie und Unfallchirurgie noch die Anästhesie anwesend.

## Diskussion

Die vorliegende Studie untersucht die Versorgungsrealität der fachlichen und personellen Zusammensetzung von Schockraumteams nach vorangegangener Schockraumalarmierung in 11 ÜTZ in Deutschland und einem ÜTZ in der Schweiz. Die Untersuchung mit prospektivem konsekutivem Patienteneinschluss zeigt, dass in 97,6 % der Fälle mindestens 5 der nach Weißbuch geforderten Teammitglieder des Schockraumbasisteams und bei fast 95 % der Schockraumversorgungen alle 4 nach Weißbuch geforderten Fachdisziplinen bei der Schockraumversorgung anwesend waren. Bei Vorliegen von Kriterien der Kategorie A für die Schockraumalarmierung nach Weißbuch waren in nahezu 98 % der Schockraumversorgungen alle geforderten Disziplinen im Schockraum anwesend. Dies bedeutet eine hohe Leitlinientreue in den teilnehmenden Studienzentren. Dies könnte ein Ausdruck eines Selektionsbias durch die teilnehmenden Kliniken selbst sein, die nur einen Teil der Versorgungslandschaft abbilden. Auch wenn eine oder mehrere Fachdisziplinen bei der Schockraumversorgung gefehlt haben, konnten wir in der vorliegenden Untersuchung keinen Einfluss auf das Versterben innerhalb von 48 h nach der Krankenhausaufnahme verzeichnen.

In der internationalen Literatur finden sich keine prospektiven vergleichenden Studien oder validierten Untersuchungen über die fachliche und personelle Zusammensetzung von Schockraumteams [13]. Betont wird häufig, dass – unabhängig von der Größe des Schockraumteams – die Zusammenstellung fester Teams, die nach vorstrukturierten Plänen arbeiten bzw. ein spezielles Training (z. B. ATLS® oder ETC®) durchlaufen haben [1, 11, 14, 19, 23], von großer Bedeutung ist. Ferner sind klinikinterne Leitlinien und Absprachen zwischen den beteiligten Fachdisziplinen [6, 18] für die prioritätenorientierte Versorgung erforderlich.

Die vorliegende Auswertung beobachtet, dass im Hinblick auf die geforderte Anwesenheit der 4 Fachdisziplinen in der absoluten Mehrheit (98 %) der Fälle keine Unterversorgung bei Vorliegen von A‑Kriterien [9] vorlag. Bei Fehlen von A‑Kriterien ist das Schockraumteam in knapp 93 % der Fälle mit allen 4 Fachdisziplinen vollständig. Bei den innerhalb von 48 h im Krankenhaus verstorbenen Patienten waren die Schockraumteams mit allen 4 Fachdisziplinen vollständig, d. h., die Mortalität lässt sich nicht über ein fehlendes Teammitglied bzw. eine fehlende Disziplin während der Schockraumversorgung erklären. Der geringe Unterschied in der initialen Teamgröße bei Vorliegen von A‑ bzw. B‑Kriterien kann anhand der vorliegenden Daten nicht erklärt werden. Zu berücksichtigen ist möglicherweise die Tatsache, dass die Schockraumanmeldungen in den Kliniken föderal unterschiedlich geregelt sind. So wird z. B. in Bayern seit Ende 2018 mit Beschluss des Rettungsdienstausschusses Bayern und des Bayerischen Innenministeriums [17] präklinisch die Unterscheidung zwischen Schockraum A (offensichtliche schwere Verletzung und/oder Trauma mit Störung von Vitalparametern) und Schockraum B (potenziell schwer verletzter Patient aufgrund des Unfallmechanismus und/oder Einschätzung des präklinischen Teams zum Zeitpunkt der Anmeldung ohne offensichtliche schwere Verletzung und mit stabilen Vitalparametern) gemacht und bei der Anmeldung über die integrierten Leitstellen entsprechend angegeben. In anderen Bundesländern mag dies auf Basis informellerer Regelungen berücksichtigt werden. Möglich ist hier, dass lokale Absprachen zwischen den an der Schockraumversorgung beteiligten Kliniken bei Anmeldungen mit B‑Kriterien das Team erst nach der Erstbeurteilung bedarfsweise aufwachsen lassen. Möglich ist auch, dass in der Traumaversorgung sehr erfahrene Kollegen der Orthopädie und Unfallchirurgie die initiale Versorgung in einem kleineren Team vornehmen, um die Ressourcen (z. B. Assistenzarzt Orthopädie und Unfallchirurgie, Anästhesie, MTRA) zur Versorgung der anderen Notfallpatienten zu schonen. Forderungen nach einem „Schockraum-Light-Team“ werden aufgrund steigender Schockrauminanspruchnahmen und des erheblichen Vorhalteaufwands [15] diskutiert [3]. Aufgrund des beobachtenden Studiendesigns kann aus der vorliegenden Studie auf Basis der Daten keine Ableitung bezüglich der Empfehlung für ein „Schockraum-Light-Team“, d. h. den Einsatz eines reduzierten Basisteams für die Schockraumversorgung bei Fehlen von A‑Kriterien, ausgesprochen werden. Unter dem aktuellen Erkenntnisstand muss von einer auf lokalen Absprachen basierenden, etablierten „good clinical practice“ ausgegangen werden.

Die aktualisierte S3-Leitlinie empfiehlt den Einsatz fester Schockraumteams mit fester Struktur und nach speziellem Training mit einem hohen Empfehlungsgrad [7] und betont, dass nach wie vor keine validierten Untersuchungen oder prospektive vergleichenden Studien zur Zusammensetzung der Teams vorliegen. Als Expertenkonsensus („good clinical practice point“) wurde die Empfehlung ausgesprochen, dass das interprofessionelle Schockraumteam aus mindestens 2 Pflegekräften und mindestens 2 Ärzten, die die notfallmedizinische und notfallchirurgische Kompetenz abbilden, bestehen soll [7]. Diese Expertenempfehlung, die in Analogie auch im Weißbuch für die Versorgung des kritisch kranken, nichttraumatologischen Schockraumpatienten [2] empfohlen wird, berücksichtigt die Entwicklung in der klinischen Akut- und Notfallmedizin und deren Konsolidierung mit der Zusatzbezeichnung [5]. Infolgedessen wird eine zunehmende Anzahl innerklinischer Notfallmediziner, die immer in der Notaufnahme anwesend und speziell geschult sind, im eigentlichen Spektrum der geforderten Aufgabe [7] – Sichtung, Diagnostik, Stabilisierung zu operativer und/oder intensivmedizinischer Weiterversorgung – diese erfüllen. Jedoch muss an dieser Stelle die aktuelle Richtlinie des Gemeinsamen Bundesausschusses zur Begutachtung des Medizinischen Dienstes gemäß der MD-Qualitätskontrolle-Richtlinie [16] benannt werden, die sich zu Regelungen des Gemeinsamen Bundesausschusses zu einem gestuften System von Notfallstrukturen in Krankenhäusern gemäß § 136c Absatz 4 des Fünften Buches Sozialgesetzbuch (SGB V) hinsichtlich der Schwerverletztenversorgung explizit auf die 2. Auflage des *Weißbuch Schwerverletztenversorgung* [10] bezieht und nicht auf die aktuelle Auflage [9]. In der Auflage von 2012 wird die Anwesenheit des Facharztes für Radiologie bzw. Weiterbildungsassistent mit Facharztstandard im Basisteam des ÜTZ als Grundlage der strukturellen Erfüllung festgelegt, womit im Basisteam die Anwesenheit von 8 Personen zu jeder Tages- und Nachtzeit festgelegt ist. Da die meisten zertifizierten Traumazentren in Deutschland auch an der Notfallversorgung am gestuften System von Notfallstrukturen in Krankenhäusern gemäß § 136c Absatz 4 des Fünften Buches Sozialgesetzbuch (SGB V) teilnehmen, ergibt sich hier möglicherweise eine relevante Diskrepanz zwischen den zu erfüllenden Strukturkriterien zur Teilnahme an der Notfallversorgung und der Teilnahme an der Schwerverletztenversorgung, da hier die aktuelle Zertifizierung auf Grundlage des Weißbuch 2019 erfolgt [9]. Dies gilt es in der hier geführten Diskussion, aber auch in den lokalen Planungen zu berücksichtigen.

### Limitationen

Die Stärke der vorliegenden Untersuchung ist v. a. im prospektiven konsekutiven Einschlusses aller Traumapatienten in den teilnehmenden Studienzentren zu sehen und muss, im Gegensatz zu den selektierten Patientenerfassungen im TraumaRegister der DGU®, als Maßstab angesehen werden, da auch die Schockraumpatienten mit leichten Verletzungen (Nichtbasiskollektiv des TraumaRegister DGU®) in die Auswertung eingeschlossen wurden. Im Vergleich zu der hier beschriebenen Mortalität von 3 % liegt die Mortalität in den Auswertungen des TraumaRegister DGU® im Basiskollektiv bei 11 % über die letzten 10 Jahre [20]. Da die RISC-Prognose in der vorliegenden Auswertung nicht berechnet wurde, muss dies als Limitation für den Vergleich der verschiedenen Mortalitäten der untersuchten Kohorte mit den Daten aus dem TraumaRegister DGU® gesehen werden. Ebenso werden die Interpretation und der Vergleich des mittleren ISS in der hier beschriebenen Kohorte (10,6), der deutlich unter dem des TraumaRegister-DGU®-Basiskollektivs von 18,6 Punkten liegt, durch die fehlende RISC-Prognostizierung limitiert.

Eine weitere Limitation stellen lokale Besonderheiten in der Zusammensetzung des Basisteams dar. So sind in 3 der teilnehmenden Kliniken Fachärzte für Allgemeinchirurgie mit der nötigen Erfahrung in der Versorgung schwer verletzter Patienten in der jeweiligen Dienststruktur als Facharzt im Basisteam eingesetzt. Diese erfüllen nach den klinikinternen Vorgaben die Qualifikation gemäß Weißbuch, auch wenn die formale Facharztanerkennung als Facharzt für Orthopädie und Unfallchirurgie nicht vorlag. In der vorliegenden Auswertung wurde die Anwesenheit der Fachabteilung Orthopädie und Unfallchirurgie durch den dann anwesenden Weiterbildungsassistenten bzw. Facharzt gewertet. Die vorliegenden Daten lassen keine Rückschlüsse auf die tatsächliche klinische Expertise der Teammitglieder zu, unabhängig von deren Qualifikationen aus speziellen Trainings, die durch die Zertifizierung gefordert sind (z. B. ATLS®). Ein mögliches Bias mit entsprechender Limitation bei der Interpretation der Ergebnisse mag hier vorliegen, da an der Studie ausschließlich ÜTZ mit einer hohen Motivation zur Studienteilnahme teilgenommen haben.

Zu berücksichtigen ist, dass der Patienteneinschluss zwischen Dezember 2019 und Februar 2021 erfolgte und somit teilweise von der ersten und zweiten Welle der COVID-19-Pandemie in Europa betroffen war. Durch die mit der Pandemie verbundenen Ausgangsbeschränkungen, Homeoffice-Bestimmungen und Kurzarbeit kam es deutschlandweit aufgrund der herabgesetzten Mobilität zu deutlich weniger Traumaereignissen, insbesondere Verkehrsunfällen [21]. Dadurch kann es zu einer Veränderung an Menge und Art der Schockraumzuweisungen gekommen sein. Da die vorliegende Untersuchung aber auf die Zusammensetzung des Schockraumteams fokussiert, dürfte das die Ergebnisse nicht wesentlich beeinflusst haben. Ferner lassen die Daten keine Aussage darüber zu, ob es z. B. durch COVID-19-erkrankungsbedingte Personalengpässe zu einer veränderten Teamzusammensetzung und Schockraumpräsenz gekommen ist. Allerdings spricht die sehr hohe Rate der Einhaltung der Empfehlungen aus dem Weißbuch dafür, dass trotz dieser Widrigkeiten das erforderliche hohe Niveau selbst in dieser Phase eingehalten werden konnte.

Zudem hat die Studie nicht das Kompetenzniveau hinsichtlich der Notfalleingriffe beim eingesetzten Personal – wie sie in der aktuellen S3-Leitlinie empfohlen werden – erfasst.

## Ausblick

Die hier dargestellte Versorgungsrealität von 12 ÜTZ in Deutschland und der Schweiz zeigt, dass die Anforderung in Bezug auf die Größe des Schockraumteams und die anwesenden Disziplinen umgesetzt werden. Die Empfehlungen der aktuellen „S3-Leitlinie Polytrauma/Schwerverletzten-Behandlung“ [7] scheinen sogar übererfüllt. Zwingend ist, dass die Qualifikation der Teammitglieder den Anforderungen aus der Leitlinie entspricht und die Eskalationsstufen der erweiterten Schockraumteams tatsächlich eingehalten werden. Gerade bei einer reduzierten Zahl an Personen ist die formale (ATLS® bzw. ETC® Schulung) und klinische Expertise der kleineren Teams eine entscheidende Voraussetzung. Vor dem Hintergrund zunehmender Knappheit personeller Ressourcen auch in den überregionalen Versorgungsstufen ergeht daraus die Forderung an die Klinikleitungen, entsprechende Expertise durch Ausbildungs- und Förderprogramme zur Verfügung zu stellen und die vorhandene Expertise durch Karriereentwicklungen und -perspektiven und Verbindlichkeiten bei Arbeits- und Dienstbedingungen zu binden. In der Aktualisierung des Weißbuchs muss v. a. eine klare Definition der Expertise und klinischen Qualifikation der Schockraumteams erfolgen sowie die Diskrepanz zu den neuen Empfehlungen der S3-Leitlinie und den gesetzgeberischen Strukturvorgaben berücksichtigt und angeglichen werden. Für eine valide Beurteilung der erforderlichen Expertise und Anzahl von Teammitgliedern sind Outcome-orientierte Studien erforderlich, die an einer entsprechend großen Patientenzahl über alle Versorgungsstufen hinweg valide Aussagen über die erforderliche fachliche und personelle Zusammensetzung von Schockraumteams zulassen.

## Fazit für die Praxis

- In praktisch allen Schockraumversorgungen besteht das Basisschockraumteam aus 4 Disziplinen: Orthopädie und Unfallchirurgie, Anästhesie, Radiologie und Notaufnahmepflege.
- Bei Vorliegen von Schockraumkriterien der Kategorie A gemäß dem Weißbuch erfolgt die Schockraumversorgung nahezu immer in der geforderten fachlichen und personellen Teamzusammensetzung.

## Data Availability

Die erhobenen Datensätze können auf begründete Anfrage in anonymisierter Form beim korrespondierenden Autor angefordert werden.
